# The Effects of Herbicides Targeting Aromatic and Branched Chain Amino Acid Biosynthesis Support the Presence of Functional Pathways in Broomrape

**DOI:** 10.3389/fpls.2017.00707

**Published:** 2017-05-04

**Authors:** Evgenia Dor, Shmuel Galili, Evgeny Smirnov, Yael Hacham, Rachel Amir, Joseph Hershenhorn

**Affiliations:** ^1^Department of Phytopathology and Weed Science, Institute of Plant Protection, Agricultural Research Organization, Newe Ya’ar Research CenterRamat Yishay, Israel; ^2^Institute of Plant Sciences, Agricultural Research Organization, The Volcani CenterRishon LeZion, Israel; ^3^MIGAL – Galilee Technology CenterKiryat Shmona, Israel

**Keywords:** acetolactate synthase (ALS), amino acid, broomrape, enolpyruvylshikimate phosphate synthase (EPSPS), glyphosate, imazapic

## Abstract

It is not clear why herbicides targeting aromatic and branched-chain amino acid biosynthesis successfully control broomrapes—obligate parasitic plants that obtain all of their nutritional requirements, including amino acids, from the host. Our objective was to reveal the mode of action of imazapic and glyphosate in controlling the broomrape *Phelipanche aegyptiaca* and clarify if this obligatory parasite has its own machinery for the amino acids biosynthesis. *P. aegyptiaca* callus was studied to exclude the indirect influence of the herbicides on the parasite through the host plant. Using HRT – tomato plants resistant to imidazolinone herbicides, it was shown that imazapic is translocated from the foliage of treated plants to broomrape attachments on its roots and controls the parasite. Both herbicides inhibited *P. aegyptiaca* callus growth and altered the free amino acid content. Blasting of *Arabidopsis thaliana* 5-enolpyruvylshikimate-3-phosphate synthase (EPSPS) and acetolactate synthase (ALS) cDNA against the genomic DNA of *P. aegyptiaca* yielded a single copy of each homolog in the latter, with about 78 and 75% similarity, respectively, to *A. thaliana* counterparts at the protein level. We also show for the first time that both EPSPS and ALS are active in *P. aegyptiaca* callus and flowering shoots and are inhibited by glyphosate and imazapic, respectively. Thus leading to deficiency of those amino acids in the parasite tissues and ultimately, death of the parasite, indicating the ability of *P. aegyptiaca* to synthesize branched-chain and aromatic amino acids through the activity of ALS and EPSPS, respectively.

## Introduction

Broomrapes (*Orobanche* and *Phelipanche* spp.) are weedy holoparasitic plants that parasitize the roots of many broadleaf crops and cause tremendous losses in yield and quality worldwide (Gressel and Joel, 2013). Today, herbicides are the main strategy used to control broomrape, but they have several drawbacks (Joel et al., 2007). To date, only herbicides that block the production of amino acids have been found to be effective in controlling broomrape. These include glyphosate and imidazolinones, and sulfonylureas. Glyphosate inhibits the enzyme EPSPS (EC 2.5.1.19) in the aromatic amino acid-biosynthesis pathway (Bentley, 1990; Roberts et al., 1998, 2002; Schönbrunn et al., 2001). The imidazolinones and sulfonylureas inhibit the enzyme ALS (EC 4.1.3.18) in the branched-chain amino acid-biosynthesis pathway (Duggleby et al., 2008; Eizenberg et al., 2013). Inhibition of aromatic or branched-chain amino acid synthesis restricts the plant’s ability to produce functional proteins and essential metabolites derived from those amino acids. This eventually leads to plant death.

The mode of action of herbicides that are able to control the Orobanchaceae is not known (Eizenberg et al., 2013). It is generally assumed that holoparasites such as broomrapes are not capable of synthesizing amino acids, as they lack nitrate reductase activity (Stewart et al., 1984; Press et al., 1986) and there is complete absence of glutamine synthetase, as measured in *Orobanche cernua, Orobanche hederae, Orobanche minor*, and *Phelipanche ramosa* (McNally et al., 1983). This hypothesis is supported by the observation that holoparasites can get most or all of their nitrogen in fully reduced forms, such as ammonium or amino acids (Westwood, 2013). Indeed, transfer of ^15^N_2_-labeled glutamine from *Brassica napus* to *P. ramosa* tubercles has been shown (Gaudin et al., 2014). Evidence of amino acid transport from the host to the parasite has also been reported (Aber et al., 1983; Abbes et al., 2009). There are a few reports of highly limited growth of broomrape tissue culture without an amino acid source (Ben-Hod et al., 1991). It has been proposed that aside from inhibiting EPSPS, glyphosate may also inhibit the translocation of assimilates from source leaves to various sinks (Geiger and Bestman, 1990; Geiger et al., 1999). Nadler-Hassar et al. (2004) showed that glyphosate application on the obligate parasite *Cuscuta campestris* results in reduced ^14^C-labeled sucrose and green fluorescent protein accumulation in the parasite organs. They hypothesized that the parasite’s growth is inhibited by assimilate starvation, rather than by direct herbicide inhibition of its EPSPS. However, other scientists have indicated that *Striga* and *Phelipanche* can grow and develop on minimal media tissue culture, which contains ammonium (Deeks et al., 1990; Zhou et al., 2004; Fernandez-Aparicio et al., 2011). In addition, there are indications of *de novo* amino acid synthesis in the parasite. Using ^15^N-labeled ammonium in *Orobanche ramosa*, researchers showed that tubercles assimilate inorganic nitrogen when supplied directly through batch incubation (Gaudin et al., 2014). Broomrapes attached to the roots of transgenic tobacco with target-site resistance to chlorsulfuron and to the roots of transgenic oilseed rape with target-site resistance to glyphosate were successfully controlled with chlorsulfuron and glyphosate, respectively (Joel et al., 1995). This also suggests the presence of amino acid biosynthesis in the parasite. Recently, shikimate accumulation and a decrease in free aromatic amino acids have been shown in Egyptian broomrape [*Phelipanche aegyptiaca* (Pers.) Pomel] attached to the roots of glyphosate-resistant tomato following foliar glyphosate application (Shilo et al., 2016). This suggests the presence of active EPSPS in parasite tissues. However, shikimate accumulation cannot be used as direct proof of EPSPS inhibition. The shikimate pathway includes seven different enzymes catalyzing the conversion of erythrose 4-phosphate and phosphoenol pyruvate to chorismate, which is used not only in the production of aromatic amino acids, but also in the biosynthesis of many other metabolites: vitamin K and metal chelators, ubiquinone and *p*-aminobenzoic acid, as well as many secondary metabolites, including flavanones and naphthoquinones (Roberts et al., 2002). Therefore, only the finding of EPSPS activity in *P. aegyptiaca* may conclusively solve the question of this enzyme’s presence in the Orobanchaceae.

The objectives of the present study were to elucidate the mechanisms by which glyphosate and imazapic control *P. aegyptiaca* on tomato and verify the presence and role of EPSPS and ALS enzymes in the metabolism of the parasite. Determination of herbicides’ modes of action in controlling obligate weedy parasites is not a trivial task. The anatomical and physiological connections between the host and the parasite make them, in many aspects, one organism (Rubiales et al., 2009; Yoder and Scholes, 2010). Any physiological or biochemical factor measured in one of them may result from its partner. In this study, we tackled this problematic issue by using parasite tissue culture. Although tissue culture does not always provide a true representation of the processes occurring in the host–parasite association, our tissue culture results were considerably strengthened by those obtained from the parasite attached to its host. In addition, using the HRT tomato plants, resistant to ALS-inhibiting herbicides, allowed us to restrict indirect influence of the herbicide on the parasite through the host plant.

Glyphosate has been shown to be translocated from the foliage of treated host plants to broomrape attachments on their roots (Arjona-Berral et al., 1990; Nandula et al., 1999). However, with respect to ALS-inhibiting herbicides, accumulation of radioactivity in sunflower broomrape following application of ^14^C-labeled imazapyr has been reported (Dıaz-Sanchez et al., 2002), but this cannot be used as a direct indication of the transfer of non-metabolized herbicide molecules to the parasite. Therefore, first, imazapic applied to the foliage was shown to be taken up by *P. aegyptiaca* and to inhibit its development. Then, the influence of imazapic and glyphosate on parasite tissue culture was studied; finally, activity of ALS and EPSPS enzymes in *P. aegyptiaca* tissue culture and flowering shoots, as well as inhibition of these enzymes by imazapic and glyphosate, respectively, was shown. Neither the biosynthesis of amino acids by broomrapes nor the presence of active EPSPS or ALS enzymes in the parasite has ever been demonstrated.

## Materials and Methods

### Plant Materials and Growth

Tomato (*Solanum lycopersicon*) cv. M82 seeds were obtained from Tarsis Agricultural Chemicals Ltd., Israel. HRT, a tomato mutant that is highly resistant to imidazolinone herbicides, was obtained by ethyl methanesulfonate (EMS) mutagenesis (Dor et al., 2016). Broomrape seeds were collected from Egyptian broomrape inflorescences parasitizing tomato grown in Kibbutz Beit Ha’shita, Israel. Broomrape seeds were stored in the dark at 4°C until use. Tomato plants were grown in 2-l pots using medium-heavy clay–loam soil containing broomrape seeds at a concentration of 15 ppm (15 mg seed kg^-1^ soil, ∼2250 seed kg^-1^) as described in Dor et al. (2016).

### Influence of ALS Inhibitors Applied to HRT Leaves on *P. aegyptiaca* Attached to the Roots

Imazapic and imazapyr (38.4 g a.i. ha^-1^) were applied on HRT plants—tomato mutants that are highly resistant to imidazolinone herbicides (Dor et al., 2016). Non-treated plants were used as a control. Once a week, starting 1 week after treatment for 4 successive weeks, the number of aboveground broomrape shoots was counted. The number and biomass of the broomrapes attached to the roots were recorded until the end of the experiment. Samples of tomato roots and broomrape shoots were taken for free amino acid determination 2 weeks after treatment.

### Extraction, Derivatization, and Amino Acid Analysis

Free amino acids were extracted from 100 mg plant tissue. The single-ion mass method was used for soluble amino acid determination with the RXI-5-Sil MS capillary column (RESTEK; 30 m, 52.0-mm i.d., and 0.25-mm thickness). All analyses were carried out on a GC–MS system (Agilent 7890A) coupled with a mass selective detector (Agilent 5975c) and a Gerstel multipurpose sampler (MPS2) (Cohen et al., 2014). Peak finding, peak integration, and retention-time correction were performed with the Agilent GC/MSD Productivity ChemStation package^1^. Peaks areas were normalized to an integral standard (norleucine) signal (Hacham et al., 2016).

### Imazapic Detection in Treated HRT Foliage, Roots and Broomrape

When young broomrape tubercles of 4–5 mm diameter were detected on the roots of HRT plants (6 weeks after planting), the surface of other pots at the same developmental stage was covered with cardboard to prevent any contact between the imazapic and the broomrape in the soil other than through the plant foliage, and the plants were sprayed with 20 g a.i. ha^-1^ imazapic. Non-treated plants were used as controls. Two weeks after herbicide application, the root, leaf and broomrape samples were taken for analysis of imazapic contents. Imazapic extraction and analysis were conducted by two different methods. In the first experiment we used a modified method of Krynitsky (1999) and Huang et al. (2009). Briefly, 20 g of sample was homogenized in 100 ml solution made up of 70% (v/v) 0.1 M NH_4_HCO_3_ pH 5 and 30% (v/v) methanol, with an Ultra-Turrax T25 homogenizer (Janke & Kunkel, Staufen, Germany) running at 20,500 rpm for 3 min. The filtrate volume was reduced to 50 ml by evaporation at 37°C under reduced pressure (BUCHI Rotavapor, Labortechnik GmbH, Essen, Germany). The extracts were lyophilized (Christ Alpha 1-4 LOC1 Freeze Dryer, Martin Crist, Osterode, Germany) and resuspended in dichloromethane. After filtration, the extracts were evaporated to dryness. The dry extracts were resuspended in 0.5 ml of a solution of 0.1% (v/v) formic acid in 20:50:30 acetonitrile:MeOH:H_2_O (v/v) and the imazapic content was detected by ultra-performance liquid chromatography (UPLC)–MS analysis in an Agilent 1290 Infinity series liquid chromatograph coupled with an Agilent 1290 Infinity DAD and Agilent 6224 Accurate Mass Time of Flight (TOF) mass spectrometer (Agilent Technologies, Santa Clara, CA, USA) using a Zorbax Extend-C18 Rapid Resolution HT column (2.1 mm × 50.0 mm, 1.8 μm, Agilent Technologies, Waldbronn, Germany). The gradient-elution mobile phase consisted of 95% eluent A (0.1% formic acid) and 5% eluent B (acetonitrile containing 0.1% formic acid). After 1.5 min, eluent B was increased from 5 to 60% over 5 min, and then increased from 60 to 95% over 7 min, kept at 95% for 2 min and then restored to 5% over 10 min. The flow rate was 0.3 ml min^-1^ and the column temperature was set to 40°C.

Eluted compounds were subjected to a dual-sprayer orthogonal electrospray ionization (ESI) source with one sprayer for analytical flow and one for the reference compound (Agilent Technologies, USA). The ESI source was operated in positive mode at the following settings: gas temperature of 350°C with a flow of 10 l min^-1^ and nebulizer set to 40 psig, VCap set to 4000 V, the fragmentor to 140 V and the skimmer to 65 V. Scan mode of the mass detector was applied (100–1700 m/z) at a rate of 3 spectra s^-1^. The [M+H] ions of imazapic (276.1342 Da) were detected and analyzed by Masshunter qualitative and quantitative analysis software version B.05.00 (Agilent Technologies). Quantification was calculated from an imazapic standard curve (Adama Agricultural Solutions Ltd., Ashdod, Israel).

In the second experiment, samples (500 g) were sent to Bactochem Feller Group Holdings Israel (Ness-Ziona, Israel) for imazapic determination by QuEChERS method (Lehotay et al., 2011; Wilkowska and Biziuk, 2011). Briefly, samples were ground and extracted with acetonitrile mixed with 1% (v/v) acetic acid. After centrifugation at 3500 rpm for 5 min, the supernatant was dried with MgSO_4_ and passed through Primary Secondary amine (Bondesil), dried again with MgSO_4_, mixed with water and analyzed by LC–MS/MS. Imazapic was quantified by calibration conducted with an imazapic standard.

### Injection of ALS-Inhibiting Herbicides into Young *P. aegyptiaca* Shoots

Acetolactate synthase-inhibiting herbicides were injected directly into young broomrape shoots (1 cm and 2–4 mm in diameter) emerging above the soil. The plants were injected with 5 μl water (control), or 5 μl water containing 10 nmol imazamox, imazapic, imazapyr or sulfosulfuron. The injected broomrape shoot height was evaluated on a daily basis.

### *P. aegyptiaca* Tissue Culture

Surface-disinfected *P. aegyptiaca* seeds were germinated in a 45-mm diameter Petri dish as described in Dor et al. (2007). Germinated seeds were gently transferred to solid callus growth culture medium (CGM) containing 3.1 g l^-1^ Gamborg salts, 1 ml l^-1^ B5 vitamins, 1 mM MgCl_2_, 1.5 mM CaCl_2_, 5 g l^-1^ phytagel, 600 mg l^-1^ casein hydrolysate (amicase), 30 g l^-1^ sucrose, 1μM 2,4-D, 20 μM GA_3_, 1 mg l^-1^ zeatin, pH 5.8, in 90-mm Petri dishes (modified Zhou et al., 2004). The dishes were kept in the dark at 25°C for 21 days. Then induced callus was transferred to fresh medium.

Five callus pieces, each 3 mm in diameter and about 8 mg, were placed on 45-mm Petri dishes containing CGM or the same medium without casein hydrolysate (BCGM), and kept in the dark at 25°C for 4 weeks. The callus from each dish was then weighed and biomass accumulation was calculated.

### Effects of ALS Inhibitors and Glyphosate on *P. aegyptiaca* Callus Growth

Ten callus pieces with initial biomass of 5–6 mg were placed on solid BCGM in a 90-mm Petri dish. In the imazapic experiment, a water–imazapic solution (20 μl) consisting of 0, 0.01, 0.05, 0.1, 0.5, 1, 5, or 10 μM imazapic was passed through a 0.45-μm membrane (Whatman) onto each callus piece. In the glyphosate experiment, glyphosate was embedded in BCGM at concentrations of 0.01, 0.05, 0.1, 0.5, 1, 5, or 10 μM. Dishes were kept in the dark at 25°C for 4 weeks in the imazapic experiment and for 8 weeks in the glyphosate experiment. Then the calluses from each dish were weighed to calculate biomass accumulation. The callus was immediately frozen in liquid nitrogen and stored at -80°C. Free amino acid content was analyzed in the calluses of all treatments (Hacham et al., 2016).

In the glyphosate experiment, shikimic acid accumulation (according to Zelaya et al., 2011) in the callus was analyzed. Both experiments were conducted with eight replicates (Petri dishes) per treatment.

### Shikimic Acid Determination in Liquid BCGM

Glyphosate was added to liquid BCGM to a final concentration of 5 μM. Medium without glyphosate was used as a control. About 200 mg of *P. aegyptiaca* callus (20 callus pieces) was placed in each Petri dish. Plates were kept in the dark at 25°C with shaking at 100 rpm on a rotary shaker (GFL 3017, Gesellschaft für Labortechnik mbH, Hanover, Germany) and after 4, 8, and 12 days, the callus from three Petri dishes (about 1 mg) was frozen in liquid nitrogen and stored at -80°C. Shikimic acid accumulation was analyzed in the callus and in the growth medium according to Zelaya et al. (2011). The experiment was conducted with three replicates per treatment.

### Determination of ALS and EPSPS Activities

Acetolactate synthase and EPSPS activities were determined *in vitro* using partially purified enzyme extracts from *P. aegyptiaca* callus and flowering shoots. These enzymes’ activities were also tested in young tomato leaves for comparison.

ALS extraction and assay were based on Ray (1984) and Veldhuits et al. (2000). Briefly, callus or tips of young flowering shoots were extracted in two volumes (w/v) of extraction buffer [100 mM potassium phosphate buffer pH 7.5, containing 10 mM sodium pyruvate, 0.5 mM MgCl_2_, 0.5 mM thiamine pyrophosphate (TPP), 10 μM flavin adenine dinucleotide (FAD), and 10% v/v glycerol]. The protein was precipitated with saturated ammonium sulfate and the enzyme was collected at 25–60% saturation by centrifugation at 3220 × *g* for 30 min at 4°C. Samples were chromatographically desalted on a PD-10 Sephadex G-25 column (GE Healthcare Bio-Sciences AB, Uppsala, Sweden) equilibrated with elution buffer (100 mM potassium phosphate buffer pH 7.5, containing 20 mM sodium pyruvate and 0.5 mM MgCl_2_). The enzymatic reaction assay was conducted in assay buffer (100 mM potassium phosphate buffer pH 7.0, containing 167 mM sodium pyruvate, 16.7 mM MgCl_2_, 1.67 mM TPP, and 16.6 μM FAD) at 37°C for 60 min. Imazapic, imazapyr, or rimsulfuron were added in the reaction mixture with the final concentrations from 0.1 to 200 μM for imidazolinones and from 0.001 to μM for rimsulfuron. The reaction was stopped by addition of 20 μl 6 N H_2_SO_4_ and incubation for 15 min at 60°C. Then 0.5 ml of 0.5% (w/v) creatine and 0.5 ml of 5% (w/v) α-naphthol, freshly prepared in 2.5 N NaOH, were added to the reaction mixture and the solution was incubated for an additional 15 min at 60°C. Absorbance was measured at 550 nm. A standard acetoin curve was used to quantify the reaction product. Total protein content was measured using the Bradford method (Bradford, 1976) with bovine serum albumin as the standard. One unit of ALS activity was expressed as millimole acetoin per milligram protein in 1 min and presented as percentage of activity in the control treatment, which contained no herbicide. The experiment was conducted with four replicates per treatment.

5-enolpyruvylshikimate-3-phosphate synthase extraction and assay were based on the methods of Eschenburg et al. (2002) and Priestman et al. (2005) with modifications. Briefly, callus or tips of young flowering shoots were extracted in two volumes (w/v) of extraction buffer (50 mM Tris–HCl pH 7.8, 1 mM EDTA, 0.1 M NaCl, 1 mM DTT, 0.1% v/v Triton X-100) with lysozyme (1 mg lysozyme g^-1^ plant tissue). The homogenate was then sonicated for 30 min at 4°C and centrifuged at 3220 × *g* for 15 min at 4°C. The proteins were precipitated with saturated ammonium sulfate and the enzyme was collected at 25–80% saturation by centrifugation at 3220 × *g* for 30 min at 4°C. The samples were chromatographically desalted on a PD-10 Sephadex G-25 column equilibrated with elution buffer (50 mM Tris pH 7.8, 1 mM DTT, 1 mM EDTA). EPSPS activity in the crude enzyme extract with shikimate as the substrate was assayed in assay buffer containing 500 mM MES (pH 5.5), 2 mM DTT with 250 mM shikimate, 1.0 mM phosphoenolpyruvate, and 100 mM NaHCO_3_. EPSPS activity with shikimate-3-phosphate (S3P) as the substrate was assayed in assay buffer containing 500 mM MES (pH 5.5), 2 mM DTT with 1 mM S3P and 0.1 mM phosphoenolpyruvate. Final glyphosate concentrations in the reaction mixture with shikimate were from 1 to 10^5^ μM, and with S3P – from 0.05 to 10^3^ μM. The enzyme was allowed to react for 15 min at 30°C. For colorimetric assay, a Phosphate Colorimetric Assay Kit (Sigma, MAK 030) was used. The change in optical density was measured at 620 nm. A standard curve of Na_3_PO_4_ was used to quantify the reaction product.

One unit of enzyme activity was expressed as millimole phosphate produced per milligram protein in 1 min. EPSPS activity was expressed as percentage of that in the control treatment, which contained no herbicide. The experiment was conducted with four replicates per treatment.

### Sequence Data Analysis

*Arabidopsis thaliana* ALS (NM_114714.2; Mazur et al., 1987) and EPSPS (CAA29828.1; Klee et al., 1987) coding sequences were blasted against the genomic DNA data derived from the Parasitic Plant Genome Project^2^. Deduced amino acid sequences were determined by DNAMAN 4.2 software. Plastid transit peptide and the first amino acid of the mature proteins were estimated with the ChloroP 1.1 Server^3^. Protein alignment and determination of percent similarity between DNA and protein sequences were performed with multiple-sequence comparison by log-expectation (Muscle)^4^.

### Statistical Analysis

The results were subjected to ANOVA by means of JMP software, version 5.0 (SAS Institute Inc., Cary, NC, USA). Data were compared by least-significant differences (LSD) on the basis of Tukey–Kramer Honestly Significant Difference test (α = 0.05), except for the data describing the influence of imazapic on amino acid content in HRT roots and in *P. aegyptiaca* attached to its roots, which were compared by LS Means Contrast test (α = 0.05). Data on the influence of imazapic and glyphosate on callus biomass, glyphosate on shikimic acid accumulation, imidazolinones on *P. aegyptiaca* ALS activity, and glyphosate on *P. aegyptiaca* EPSPS activity were computed by non-linear regressions using Sigma-Plot version 11.01 (SPSS Inc., Chicago, IL, USA). The data were arcsine-transformed before analysis. On the graphs, back-transformed means are presented. All experiments were conducted twice. These experiments were compared by Fisher *t*-test to prove homogeneity of the variances and then the data of the two experiments were combined, except for the experiment involving imazapic detection in tomato leaves, roots and attached broomrapes, where the data were not combined due to heterogeneity of variances. Results of those two experiments are therefore presented separately.

## Results

### Identifying Homologous Genes Encoding ALS and EPSPS

**FIGURE 1:**
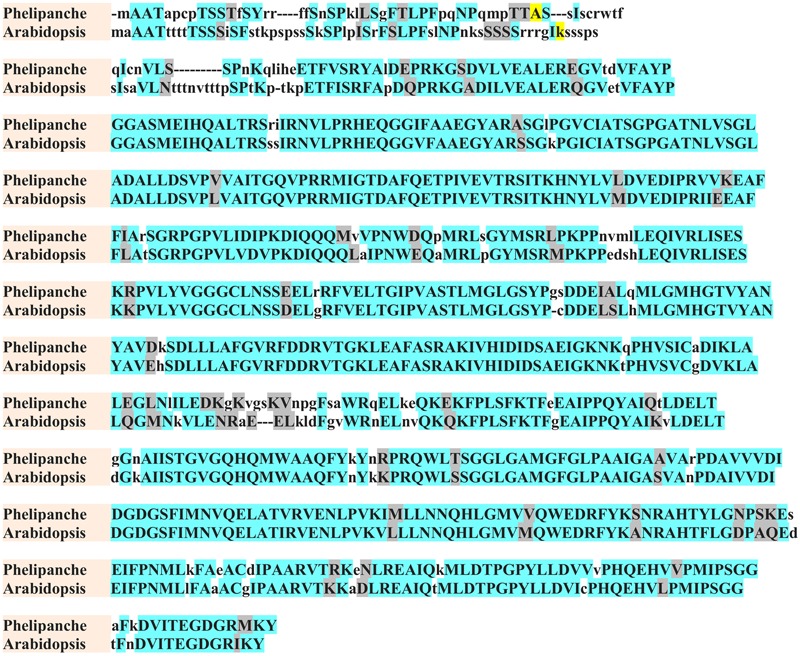
**Amino acid sequence alignment of *A. thaliana* (*Arabidopsis*) and *P. aegyptiaca* (*Phelipanche*) ALS.** Identical and similar amino acids are shown in azure and gray, respectively. Non-identical amino acids are shown in lowercase letters. First amino acids of predicted mature protein are shown in yellow.

**FIGURE 2:**
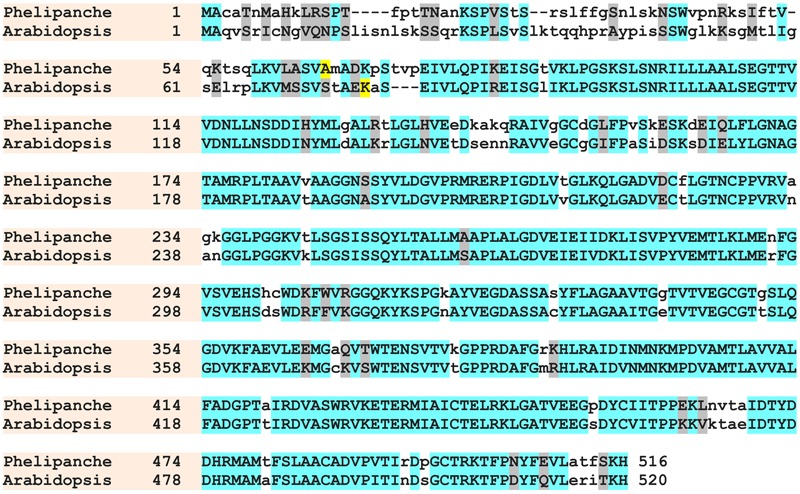
**Amino acid sequence alignment of *A. thaliana* (*Arabidopsi*s) and *P. aegyptiaca* (*Phelipanche*) EPSPS.** Identical and similar amino acids are shown in azure and gray, respectively. Non-identical amino acids are shown in lowercase letters. First amino acids of predicted mature protein are shown in yellow.

Data derived from the Parasitic Plant Genome Project^2^ identified a single DNA copy homolog of each of these enzymes as *P. aegyptiaca* putative *ALS* and *EPSPS* genes. Both of these putative genes shared about 74% homology with their corresponding *A. thaliana* genes. Predicted proteins encoded by the putative *P. aegyptiaca ALS* and *EPSPS* genes showed about 78 and 75% identity to *A. thaliana* ALS and EPSPS proteins, respectively. *P. aegyptiaca* putative ALS and EPSPS proteins both contained, as expected, a chloroplast transit peptide of 42 and 64 amino acids, respectively, at their N terminus. The predicted *P. aegyptiaca* and *A. thaliana* ALS and EPSPS mature proteins shared more than 80% sequence similarity (**Figures 1, 2** for ALS and EPSPS, respectively).

### Sensitivity of Egyptian Broomrape to ALS Inhibitors

**FIGURE 3:**
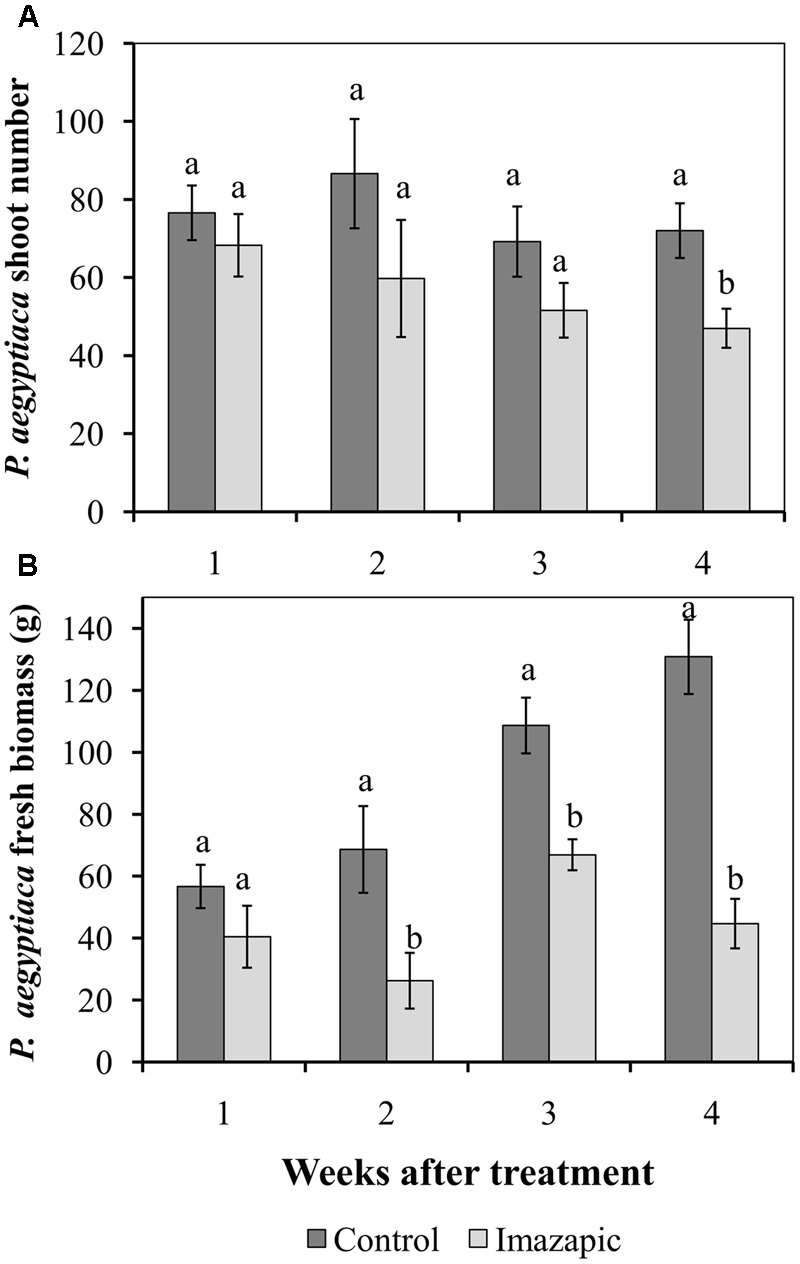
**Effect of imazapic (38.4 g a.i. ha^-1^) applied at 277 growing degree days on the number (A)** and biomass **(B)** of *P. aegyptiaca* flowering shoots attached to the roots of HRT tomato plants. Non-treated plants were used as controls. Data were recorded once a week, starting 1 week after treatment for 4 weeks. The experiment was repeated twice with 10 replicates. Results of the two experiments were compared by Fisher’s *t*-test and data were combined due to homogeneity of the variances. Results were subjected to ANOVA. Data were compared by least-significant differences (LSD), on the basis of Tukey–Kramer Honestly Significant Difference test (α = 0.05). Different letters indicate significant differences between various treatments on the same observation date. Vertical lines indicate standard errors of means (SEM).

The HRT tomato mutant was grown in soil with *P. aegyptiaca* seeds. In the control pots, *P. aegyptiaca* shoots began to emerge from the soil 44 days after planting (Supplementary Figure 1), reaching 45 ± 3.5 shoots per pot at 66 days after planting. However, at the end of the experiment, there were only 0.4 ± 0.02 *P. aegyptiaca* shoots per pot with the imazapic-treated plants, with no visible damage symptoms on the latter. The total number of broomrapes attached to HRT roots below ground was 60–80 in the first 3 weeks after herbicide application in both the control and imazapic-treated plants, with no significant difference between them. A significant reduction in the number of parasites due to imazapic was only achieved in week 4 (**Figure 3A**). However, accumulation of *P. aegyptiaca* biomass ceased from the second week after imazapic application. The total *P. aegyptiaca* biomass attached to the roots of untreated plants was 130 ± 8.1 g, as compared to 45 ± 3.7 g in the imazapic-treated plants (**Figure 3B**).

Imazapic had no effect on the levels of branched-chain amino acids in HRT roots, nor did it change their total content of soluble amino acids (**Table 1**). However, the amount of soluble Val, Leu, and Ile, as well as of total soluble amino acids, in *P. aegyptiaca* attached to treated plants was significantly reduced compared to *P. aegyptiaca* on roots of non-treated plants (**Table 1**).

**Table 1:** Effect of imazapic (38.4 a.i. ha^-1^) applied to the foliage on soluble branched-chain amino acid content (nM g^-1^ FW).

Amino acids	HRT plants		*Phelipanche aegyptiaca*	
	Control	Imazapic	Control	Imazapic
Val	105.17 ± 8.16	105.42 ± 2.98	174.94 ± 30.57	74.06 ± 4.67^∗^
Leu	160.84 ± 18.41	139.51 ± 7.54	293.54 ± 38.92	102.33 ± 2.68^∗^
Ile	102.67 ± 6.83	93.61 ± 6.89	252.29 ± 52.78	69.09 ± 4.05^∗^
Total	3698.69 ± 482.68	2792.22 ± 450.86	9710.84 ± 8809	2476.48 ± 126.71^∗^

*^∗^Significant difference between control and imazapic treatment at α = 0.05 according to LS Means Contrast test.*

Injection of ALS-inhibiting herbicides into young *P. aegyptiaca* shoots completely inhibited broomrape growth, followed by deterioration and death, with no visible effects on tomato cv. M82 plants. The water-injected shoots grew rapidly, reaching their maximal height of about 170 mm after 20 days (Supplementary Figure 2). The height of the shoots injected with the herbicides was 8.5- to 10-fold lower (10–20 mm).

### Imazapic Detection in Treated HRT Foliage, Roots and Broomrape

In both experiments, the herbicide could be found in the parasite tissue 2 weeks after the foliar application, but at concentrations that differed between the two experiments (**Table 2**). These differences might be explained by differences in the development and growth of the tomato plant and the parasite in the two experiments: not only was the method of imazapic extraction and analysis different, but the timing was as well. The first experiment was conducted in January, and the time elapsed from planting to *P*. *aegyptiaca* shoot emergence above the soil was about 2 months. At that time, the HRT plants had developed to maturity. In the second experiment, conducted in August, the first *P*. *aegyptiaca* shoots appeared above the soil after only 5 weeks, and the tomato plants at the herbicide-application stage were younger and smaller.

**Table 2:** Imazapic concentration in HRT tomato roots and leaves and in *P. aegyptiaca* attached to the roots after foliar application of the herbicide at a rate of 50 g a.i. ha^-1^.

Sample	Imazapic concentration (ng g^-1^ FW)	
	Experiment 1	Experiment 2
Leaves	196.68 ± 22.67a	480.00 ± 33.51a
Roots	12.25 ± 2.18b	0.00 ± 0.00c
*P. aegyptiaca*	4.00 ± 0.82c	280.00 ± 23.67b

*Different letters indicate significant differences in each experiment according to Tukey–Kramer Honestly Significant Difference test at α = 0.05.*

### Tissue Culture

*P. aegyptiaca* tissue culture was grown with and without amino acids: on CGM containing two forms of inorganic nitrogen (2.5 g l^-1^ nitrate in the form of potassium nitrate and 134 mg l^-1^ ammonia in the form of ammonium sulfate) and casein hydrolysate as a source of amino acids, and on BCGM containing only inorganic nitrogen. By the end of the experiment, which lasted 4 weeks, the *P*. *aegyptiaca* callus biomass had increased by 0.24 and 0.26 g per plate on CGM and BCGM, respectively. There were no significant differences in tissue culture growth rate between the two media, indicating that *P. aegyptiaca* tissue cultures can synthesize amino acids on their own.

### Influence of Imazapic on Biomass Accumulation of *P. aegyptiaca* Callus

**FIGURE 4:**
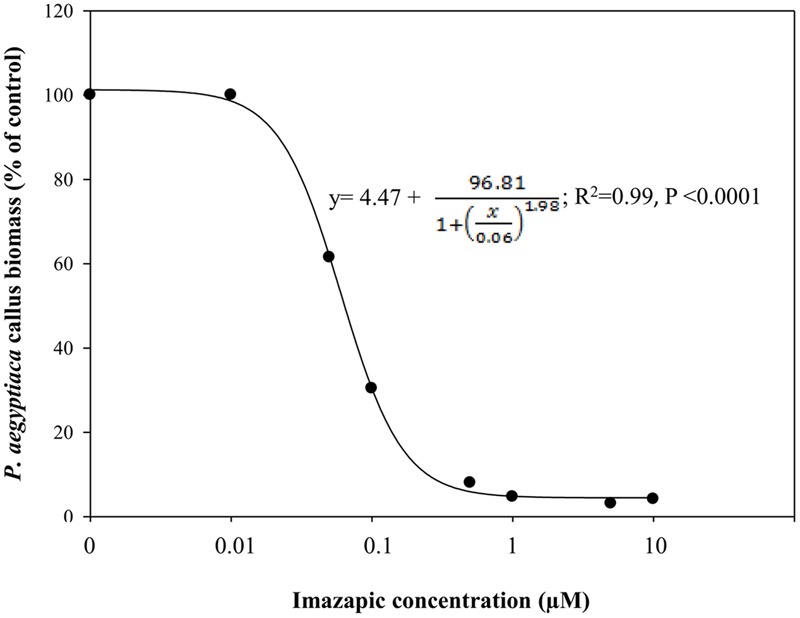
**Biomass accumulation of *P. aegyptiaca* callus grown on BCGM containing imazapic.** The experiment was conducted with eight replicates. The data were computed by non-linear regressions using Sigma-Plot version 11.01. 
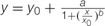
; *R*^2^ = 0.99, *P* < 0.0001, *a* = 96.81, *b* = 1.98, *x*_0_ = 0.06, *y*_0_ = 4.47. The data were arcsine-transformed before analysis. On the graph, back-transformed means are presented.

*Phelipanche aegyptiaca* tissue culture was highly sensitive to imazapic (**Figure 4**). A concentration of 0.05 μM significantly decreased biomass accumulation. ID_50_ (the herbicide concentration causing 50% growth inhibition) of imazapic was 0.06 ± 0.002 μM. Concentrations of 0.5 μM and higher completely arrested callus growth. A concentration of 10 μM imazapic resulted in blackened calluses that died. Free amino acid content increased with the increase in imazapic concentration to a maximum at 0.5 μM, and then decreased (**Table 3**). This might be the result of protein degradation, or inhibition of protein synthesis caused by a deficiency in branched-chain amino acids. Val was the most influenced by the herbicide (**Table 3**). At rates of 1–10 μM, free Val content was significantly lower than in the control. Surprisingly, the content of Glu, which is not synthesized in the branched-chain amino acids pathway, was also decreased compared to controls by the same imazapic concentrations. A general increase in total free amino acid content after herbicide treatments, which can be attributed to proteolysis, may mask the decrease in specific amino acid synthesis induced by the inhibitor (Orcaray et al., 2010). Therefore, it is useful to express the specific amino acid content as a percentage of the total free amino acids instead of in absolute values. This calculation revealed a significant reduction in soluble Val and Leu, indicating direct inhibition of the amino acid biosynthesis machinery in *P. aegyptiaca* callus (Supplementary Figure 3).

**Table 3:** Free amino acid content in *P. aegyptiaca* callus grown in imazapic-containing medium.

	Imazapic concentrations (μM)							
	0	0.01	0.05	0.1	0.5	1	5	10
	
Amino acids	Free amino acid content (nmol g^-1^ FW)							
Asp	86.9b	105ab	103ab	148a	149a	102 ab	87.2b	10lab
Met	2.0b	3.5b	3.1b	4.5ab	7.6a	2.3b	2.0b	2.4b
Lys	9.2	9.0	9.2	8.6	0.0	10.5	8.2	11.6
Thr	42.5e	67.7de	52.3e	127bc	232a	161b	125bc	94.4cd
Ile	5.4c	8.8bc	7.6bc	13.3ab	17.3a	8.0bc	6.9bc	5.6c
Leu	5.4bc	9.2ab	8.9ab	12.8a	10.5a	4.8bc	5.1bc	4.0c
Val	16.6c	36.2b	33.9b	48.9a	33.9b	7.1d	8.5d	6.3d
Phe	4.0c	6.2c	6.1c	9.0c	27.0b	18.5bc	28.1b	65.6a
Tyr	86.3b	108.2b	116b	136ab	146a	91.6b	104.2b	107.4b
Trp	3.1b	7.4b	6.4b	11.0	28.1a	7.2b	14.7ab	25.6a
Gly	13.1c	23.8b	20.1bc	23.2b	59.1a	20.6bc	20.4bc	18.3bc
Cys	1.4b	1.7b	3.0b	2.8b	8.3a	1.8b	1.9b	1.5b
Ser	73.9d	179.6bc	163bc	209b	308a	174bc	142c	193b
Pro	4.5c	11.7c	9.1c	18.2c	61.4ab	56.5ab	51.4b	69.9a
Glu	123ab	158a	143a	159a	102b	42.2c	27.3c	49.5c
Gin	58.9f	107.9e	108.6e	173.7d	637.0a	451.5b	320.7c	422.6b
Asn	52.3c	102b	83.8bc	113.2	203a	100b	168ab	149ab
Ala	59.1e	146bc	160bc	175b	385a	118cd	71de	54e
Total	649d	1096c	1041c	1394b	2421a	1379b	1194bc	1384b

*Different letters indicate significant differences between the treatments according to Tukey–Kramer Honestly Significant Difference test at α = 0.05.*

### ALS Activity in *P. aegyptiaca* and Its Response to ALS Inhibitors

**FIGURE 5:**
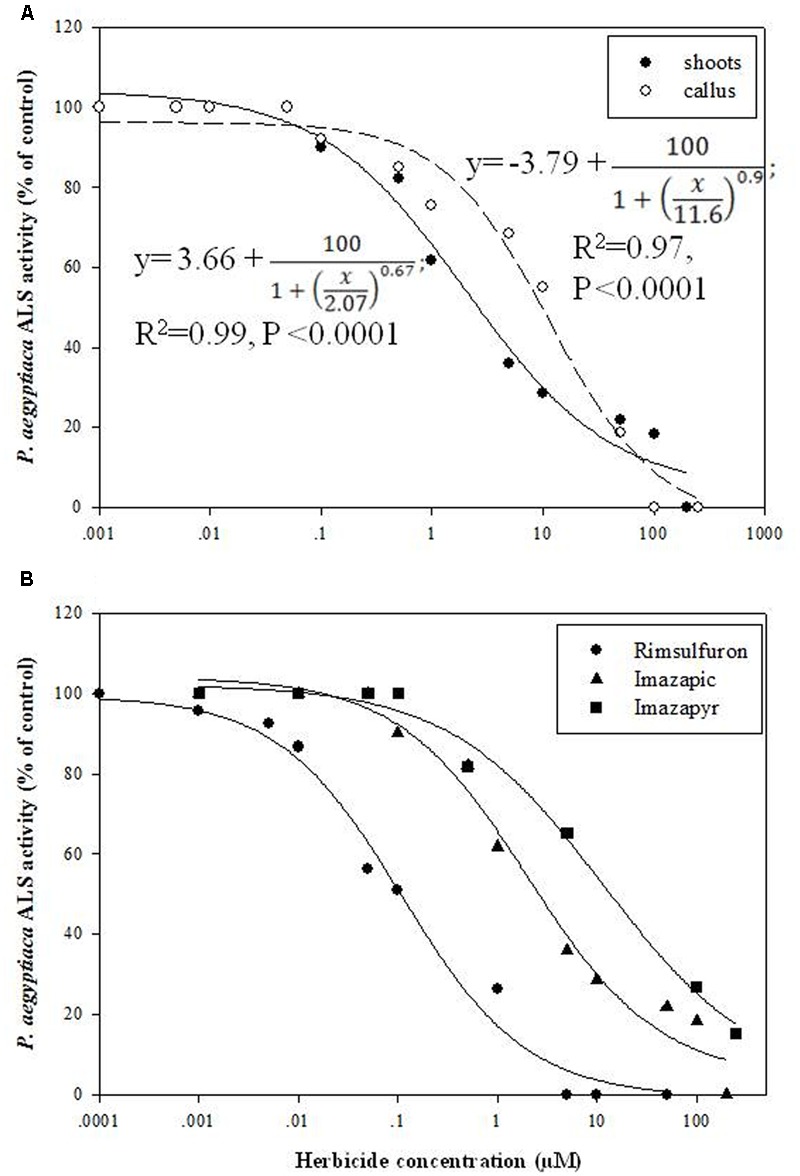
**Influence of ALS inhibitors on ALS activity of *P. aegyptiaca.* (A)** Influence of imazapic on ALS extracted from flowering shoots and callus. **(B)** Influence of imazapic, imazapyr, and rimsulfuron on ALS extracted from *P. aegyptiaca* flowering shoots. The experiment was conducted with four replicates per treatment. The data were computed by non-linear regressions using Sigma-Plot version 11.01. 
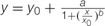
; **(A)** For flowering shoots: *R*^2^ = 0.99, *P* < 0.0001, *a* = 100, *b* = 0.67, *x*_0_ = 2.07, *y*_0_ = 3.66; for callus: *R*^2^ = 0.97, *P* < 0.0001, *a* = 100, *b* = 0.90, *x*_0_ = 11.6, *y*_0_ = -3.79. **(B)** For imazapic: *R*^2^ = 0.99, *P* < 0.0001, *a* = 100, *b* = 0.67, *x*_0_ = 2.07, *y*_0_ = 3.66; for imazapyr: *R*^2^ = 0.99, *P* = 0.0073, *a* = 100, *b* = 0.56, *x*_0_ = 11.62, *y*_0_ = 2.18; for rimsulfuron: *R*^2^= 0.98, *P* < 0.0001, *a* = 100, *b* = 0.70, *x*_0_ = 0.11, *y*_0_ = -0.61. The data were arcsine-transformed before analysis. On the graphs, back-transformed means are presented.

Enzyme extracts from *P. aegyptiaca* flowering shoots and callus both demonstrated ALS activity (3.3 and 16.6 enzyme units, respectively), similar to the level found in young tomato leaves (about 3.1 units). Accordingly, inhibition of 50% activity (ID_50_) for shoots occurred at 2 ± 0.7 μM imazapic whereas for callus, it occurred at 11.6 ± 2.6 μM (**Figure 5A**). The ID_50_ for tomato leaves was about 0.47 ± 0.1 μM, much lower than for *P. aegyptiaca* shoots. A concentration of 200 μM imazapic completely inhibited the activity of ALS from *P. aegyptiaca* shoots and callus. Comparison of the inhibitory ability of various ALS inhibitors showed that rimsulfuron (sulfonylurea) is much more potent than the imidazolinones imazapic and imazapyr (**Figure 5B**), with ID_50_ values for ALS extracted from *P. aegyptiaca* shoots of 0.11 ± 0.03, 2.07 ± 0.07, and 11.62 ± 1.2 μM, respectively.

### Influence of Glyphosate Embedded in the Growth Medium on Biomass Accumulation of *P. aegyptiaca* Callus

**FIGURE 6:**
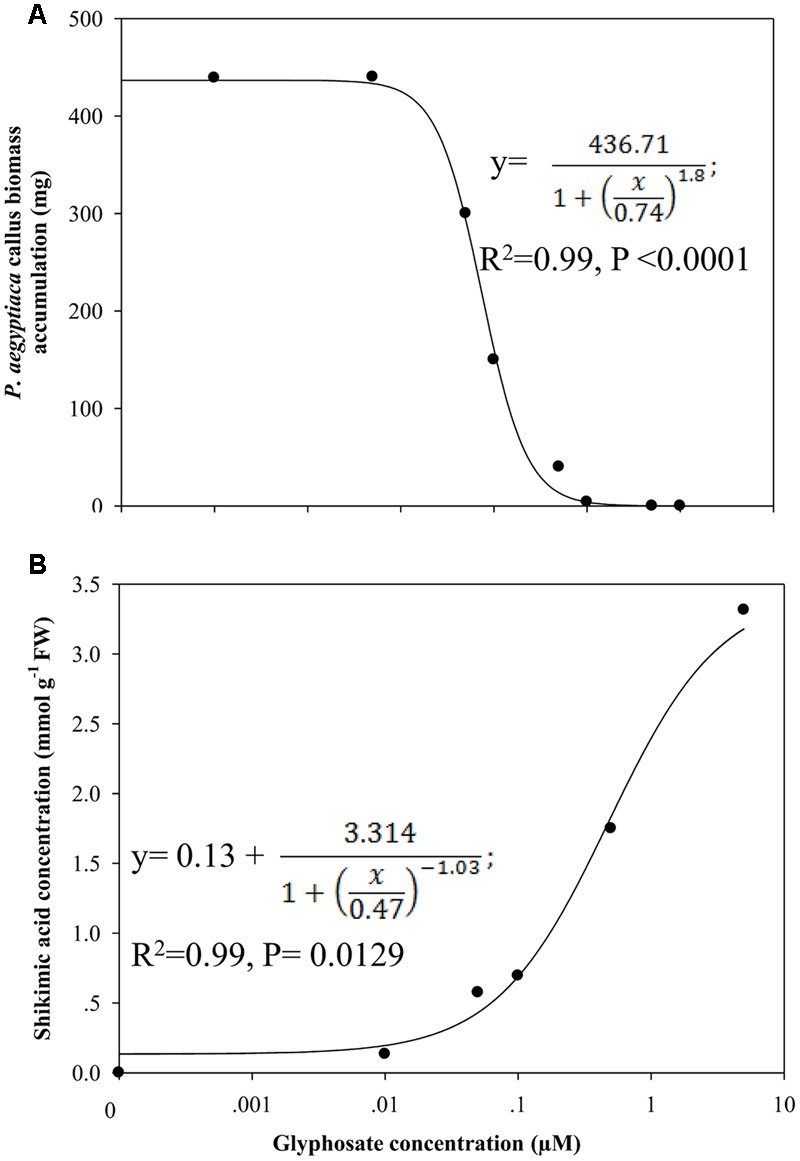
**Biomass accumulation of *P. aegyptiaca* callus (A)** and shikimic acid accumulation in the callus **(B)** cultured on BCGM containing glyphosate. The experiment was conducted with eight replicates. Data were computed by non-linear regressions using Sigma-Plot version 11.01. **(A)** For biomass: y = 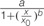; *R*^2^ = 0.99, *P* < 0.0001, *a* = 436.71, *b* = 1.80, *x*_0_ = 0.74. **(B)** For shikimic acid accumulation: 
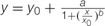
; *R*^2^= 0.99, *P* = 0.0129, *a* = 3.314, *b* = -1.03, *x*_0_ = 0.47, *y*_0_ = 0.13. Data were arcsine-transformed before analysis. On the graphs, back-transformed means are presented. FW, fresh weight.

A significant reduction in biomass accumulation was found at a concentration of 0.5 μM glyphosate (**Figure 6A**). The ID_50_ value of glyphosate was 0.74 ± 0.04 μM. Inhibition of callus growth was followed by dose-dependent shikimic acid accumulation from 0.05 to 5 μM glyphosate (**Figure 6B**). Total free amino acid content in the callus increased significantly in the presence of 0.5 μM glyphosate, suggesting inability to synthesize proteins to their full structure, leaving unused amino acids, or an indirect effect of the herbicide leading to protein degradation (**Table 4**).

**Table 4:** Free amino acid content in *P. aegyptiaca* callus grown in BCGM with embedded glyphosate.

	Glyphosate concentrations (μM)							
	0	0.01	0.05	0.1	0.5	1	5	10
	
Amino Acids	Free amino acid content (nmol g^-1^ FW)							
Asp	17.9b	16.1b	13.6b	15.3b	32.3ab	43.0a	14.8b	9.4b
Met	0.7	1.0	0.8	1.1	1.8	2.9	1.2	0.3
Lys	8.1b	9.1b	5.8b	10.8b	8.5b	32.2a	8.7b	6.9b
Thr	14.5bc	15.2bc	11.9c	16.0bc	25.4b	48.1a	31.4b	34.5ab
Ile	4.1b	3.7b	4.4b	5.1ab	5.7ab	8.3a	5.4ab	2.7b
Leu	4.2b	6.7ab	4.1b	8.0ab	6.2ab	11.1a	6.5ab	2.7b
Val	16.0c	24.4bc	19.8bc	32.5bc	41.9b	78.5a	56.9ab	17.1c
Phe	1.5a	1.0a	1.7a	1.4a	2.0a	3.7a	2.5a	0.7b
Tyr	21.1c	17.3c	29.2c	16.6c	53.6b	74.1a	70.6a	20.3c
Trp	0.4	0.5	0.7	1.2	1.1	3.2	1.5	0.6
Gly	12.4b	24.0ab	24.0ab	56.7a	35.7ab	50.7ab	35.2ab	37.0ab
Cys	2.1	1.8	2.6	2.8	5.5	8.0	5.3	1.8
Ser	21.90b	32.2b	27.3b	23.7b	144a	122a	29.9b	43.6b
Pro	3.9c	6.8bc	2.8c	5.2c	10.2b	18.3a	6.4bc	3.8c
Glu	37.7c	40.2c	37.00c	35.70c	275.6a	138.4b	26.1c	14.3d
Gln	17.1c	12.3c	14.2c	45.6c	213.5b	567a	466a	589a
Asn	7.3c	8.4c	11.1c	14.2c	34.8b	68.3a	47.4ab	13.8c
Ala	24.6d	46.763c	44.5c	42.1c	324a	357a	108b	81.0bc
Total	289e	341de	329e	408cde	1296ab	1709a	997bc	953bcd

*Different letters indicate significant differences between the treatments according to Tukey–Kramer Honestly Significant Difference test at α = 0.05.*

The levels of the soluble aromatic amino acids Phe and Trp are expected to decrease when glyphosate is applied. Surprisingly, differences in soluble Phe and Trp contents in the callus were only found at a glyphosate concentration of 10 μM, which was lethal to the callus. Calculation of the percentage of aromatic amino acids out of the total amino acid content showed decreasing levels of Phe and Trp. However, the level of Tyr was the same as in controls (Supplementary Figure 4).

In a further experiment, callus growth in liquid BCGM allowed analyzing the dynamics of shikimic acid accumulation in the tissue culture and its release into the medium as a result of callus cell deterioration. Most of the shikimic acid was found in the callus 4 days after growth initiation in medium with glyphosate (Supplementary Figure 5). After 8 days, shikimic acid level in the callus decreased and simultaneously increased in the growth medium. The callus turned brown and died after 12 days, and all of the shikimic acid was found in the medium. Cell-wall deterioration was probably the cause for the release of free amino acids from *P. aegyptiaca* callus cells into the medium. In the callus, total free amino acid content decreased significantly in the glyphosate-containing medium compared to controls (**Table 5**). A significant reduction in aromatic amino acids Phe and Trp, but not Tyr, was found. In addition, the contents of Asp, Thr, Ile (which are synthesized in the aspartate pathway) and Ser decreased, probably indicating protein degradation.

**Table 5:** Free amino acid content (nmol g^-1^ FW) in *P. aegyptiaca* callus grown in liquid BCGM with and without 5 μM glyphosate.

Amino acids	After 4 days		After 12 days	
	Control	Glyphosate 5 μM	Control	Glyphosate 5 μM
Asp	79.9	59.5	48.4	30.4^∗^
Met	5.7	4.9	4.7	4.5
Thr	148	143	173	118^∗^
Ile	20.3	22.0	31.9	21.4^∗^
Leu	10.7	13.3	28.7	21.4
Val	29.6	36.1	41.4	34.2
Phe	17.4	15.9	32.3	18.4^∗^
Tyr	241	260	253	233
Trp	15.5	7.4	60.5	25.7^∗^
Gly	30.9	32.0	77.8	45.1
Cys	5.6	7.2	13.8	10.2
Ser	229	212	215	140^∗^
Pro	52.8	55.2	70.2	48.0
Glu	71.6	42.2^∗^	52.9	40.7
Gln	485	254^∗^	249	227
Asn	39.7	19.5^∗^	32.7	18.4
Ala	438	472	380	321
Total	1925	1660	1785	1361^∗^

*^∗^Significant difference between control and glyphosate treatment at α = 0.05 according to LS Means Contrast test.*

### EPSPS Activity in *P. aegyptiaca* and Its Response to Glyphosate

Analysis of EPSPS activity in an *in vitro* assay with *P. aegyptiaca* flowering shoot extracts yielded 0.5 and 0.2 enzyme units for the substrates shikimate and S3P, respectively, and in the callus, 1.34 and 0.5 enzyme units with shikimate and S3P as substrates, respectively. In comparison, in young tomato leaves, EPSPS activity was about 0.76 and 1.05 units with shikimate and S3P as substrates, respectively.

**FIGURE 7:**
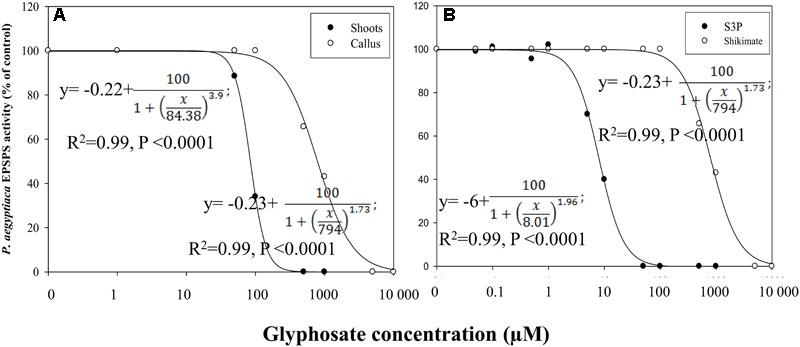
**Influence of glyphosate on EPSPS activity of *P. aegyptiaca*. (A)** Flowering shoots and callus with shikimic acid as substrate. **(B)** EPSPS activity extracted from *P. aegyptiaca* callus with shikimic acid and shikimate-3-phosphate (S3P) as substrates. The experiment was conducted with four replicates per treatment. Data were computed by non-linear regressions using Sigma-Plot version 11.01. y = y_0_ + 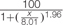; **(A)** For flowering shoots: *R*^2^ = 0.99, *P* < 0.0001, *a* = 100, *b* = 3.90, *x*_0_ = 84.38, *y*_0_ = –0.02; for callus: *R*^2^= 0.99, *P* < 0.0001, *a* = 100, *b* = 1.73, *x*_0_ = 794, *y*_0_ = –0.23. **(B)** For shikimic acid as substrate: *P* < 0.0001, *a* = 100, *b* = 1.73, *x*_0_ = 794, *y*_0_ = –0.23; for S3P as substrate: *P* < 0.0001, *a* = 100, *b* = 1.96, *x*_0_ = 8.01, *y*_0_ = –6.00. Data were arcsine-transformed before analysis. On the graphs, back-transformed means are presented.

Glyphosate inhibited EPSPS activity of both flowering shoots and callus (**Figure 7A**). EPSPS extracted from flowering shoots was much more sensitive to the herbicide than the enzyme extracted from callus, with respective ID_50_ values of about 84 ± 0.03 and 794 ± 35 μM, using shikimate as the substrate (**Figure 7A**). The enzymatic reaction with this substrate is less sensitive to glyphosate (Priestman et al., 2005) than the same reaction with the natural substrate S3P. With the latter, the ID_50_ value for EPSPS from callus was about 8 ± 0.29 μM (**Figure 7B**).

## Discussion

Holoparasites depend on their host for carbon and nitrogen resources to grow and develop (Kawachi et al., 2008; Irving and Cameron, 2009). The flow of these resources from the host to the parasite is the limiting factor for holoparasite growth (Hibberd et al., 1998, 1999). The holoparasites *Orobanche* and *Phelipanche* can only be controlled by amino acid biosynthesis-inhibiting herbicides (Duggleby et al., 2008; Eizenberg et al., 2013). This can occur via direct inhibition of an autonomic amino acid biosynthesis mechanism within the parasite, if it exists, indirectly via inhibition of branched-chain or aromatic amino acid production in the host or transport from host to parasite, or both. To distinguish among these possibilities, we first studied whether the parasite has homologous genes encoding ALS and EPSPS.

The *P. aegyptiaca* genome, similar to *Arabidopsis* and tobacco (Mazur et al., 1987), was found to contain at least one copy/genome of *ALS*, and similar to petunia and tomato, a putative *EPSPS* (Gasser et al., 1988). In addition, all conserved amino acid regions in which herbicide-resistance mutations have been found (Duggleby et al., 2008) were also present in the predicted *P. aegyptiaca* ALS protein (**Figure 1**), supporting the presence of *ALS* in *P. aegyptiaca.* The predicted *P. aegyptiaca* and *A. thaliana* ALS and EPSPS mature proteins shared more than 80% sequence similarity (**Figures 1, 2** for ALS and EPSPS, respectively). These results correspond well with the findings of Westwood et al. (2012) who reported the presence in *P. aegyptiaca* of seven target genes for ALS inhibitors and three target genes for glyphosate with about 80% homology to *Arabidopsis.*

### Sensitivity of Egyptian Broomrape to ALS Inhibitors

The presence of genes does not necessarily mean that they encode functional proteins. Thus, we sought to determine whether the encoded proteins are functional, and whether they are sensitive to herbicides. We searched for a system that would separate the effect on the host from the effect on the parasite. To reveal the role of ALS and its inhibitors in the parasite, we chose HRT, a tomato mutant that is highly resistant to imidazolinone herbicides (Dor et al., 2016). Use of this mutant enabled testing the direct effect of the herbicide on the parasite without causing any damage to the host plant. This is important because application of these herbicides on a sensitive host will block branched-chain amino acid biosynthesis in the host, resulting in a shortage of amino acids transported to the parasite and leading to its death. Thus, we demonstrated the ability of imazapic applied on HRT plants to prevent *P. aegyptiaca* shoot formation above soil level (**Figure 3** and Supplementary Figure 1), affecting the amount of soluble Val, Leu, and Ile (**Table 1**).

Imazapic had no influence on the levels of branched-chain amino acids in HRT roots, nor did it change their total content of soluble amino acids (**Table 1**). However, both were significantly reduced in *P. aegyptiaca* attached to treated plants compared to *P. aegyptiaca* on roots of non-treated plants (**Table 1**). These results may demonstrate a direct influence of imazapic on the parasite, or an effect via interruption of transport between the host and the parasite. However, translocation from host to parasite has never been reported for ALS-inhibiting herbicides, although it has been proven for glyphosate (Arjona-Berral et al., 1990; Nandula et al., 1999). Thus, we determined the level of imazapic in the leaves and roots, and in *P. aegyptiaca* attached to the roots of HRT plants treated with imazapic. Before applying the herbicide, we prevented any possible contact between the herbicide and the soil in the pots. The only way the herbicide could move to the *P. aegyptiaca* attached to its roots was via the treated HRT foliage. As this experiment was key to proving our hypothesis, we conducted two independent experiments using different analytical methods. In both experiments, imazapic applied to the foliage was detected in broomrape, proving the translocation of the herbicide from host to parasite (**Table 2**). In the three experiments involving imazapic application on HRT plants, the latter showed no visible damage symptoms.

The results to this point indicated that the herbicide is transferred from the host to the parasite and reduces the latter’s development, but has no influence on the amino acid balance, normal development or appearance of the host. These results strongly suggested that the parasite has its own active ALS that is sensitive to the herbicide. To further test this assumption, we used four herbicides belonging to two ALS-inhibiting classes: imidazolinones (imazamox, imazapic, and imazapyr) and sulfosulfuron, a sulfonylurea herbicide registered in Israel for *P. aegyptiaca* control in tomato (Dor et al., 2016). These herbicides were injected into *P. aegyptiaca* shoots that had just begun to emerge from the soil. Shoot growth was arrested, followed by deterioration and death, while water-injected shoots were not damaged (Supplementary Figure 2). To determine whether the herbicides were translocated from the parasite-injected shoots to the host, we used tomato cv. M82 plants, which are extremely sensitive to imidazolinone herbicides (Dor et al., 2016). We expected to detect visible damage symptoms on the plants if translocation does indeed occur. However, such symptoms could not be observed on the M82 plants, suggesting that the herbicide is not translocated from the parasite to the host.

To further study the assumption that the parasite has functional ALS and EPSPS, *P. aegyptiaca* tissue cultures were grown with and without amino acids: on CGM containing two forms of inorganic nitrogen and casein hydrolysate as a source of amino acids, and BCGM containing only inorganic nitrogen. By the end of the experiment, no significant differences were observed in *P. aegyptiaca* tissue culture growth rate between the two media, indicating that *P. aegyptiaca* tissue culture has the ability to synthesize amino acids on its own.

High sensitivity of *P. aegyptiaca* tissue cultures to imazapic (**Figure 4**) followed by an increase in total free amino acid content with a transient decrease in the proportion of the branched-chain amino acids Val and Leu (**Table 3** and Supplementary Figure 3) indicated direct inhibition of the amino acid biosynthesis machinery in *P. aegyptiaca* callus. Similar results were obtained by Orcaray et al. (2010) with pea plants. In addition, elevation in Thr level (**Table 3**), the main substrate for the synthesis of branched-chain amino acids in these plants (Jander and Joshi, 2010), strongly suggested considerable slowing of the pathway. A concentration of 10 μM imazapic resulted in blackened calluses that died.

The above results suggested that ALS is active in *P. aegyptiaca*. Indeed, both shoots and callus demonstrated ALS activity, similar to the level found in young tomato leaves. ALS-unrelated acetoin production in low amounts was reported by Forlani et al. (1999) in suspensions of actively proliferated cells of carrot, tobacco, maize, and rice as a side reaction of TPP dependent pyruvate-decarboxylating enzymes. Strong inhibition of acetoin production by ALS-inhibiting herbicides and a dose response curve of the inhibition of the reaction with herbicides indicate activity of ALS extracted from both shoots and callus (**Figure 5**).

### Evidence of Functional EPSPS Activity in *P. aegyptiaca*

We next sought to determine whether EPSPS is also functional in the parasite. Based on the results obtained with ALS, we expected to detect EPSPS activity in *P. aegyptiaca*, since glyphosate has been found to control *P. aegyptiaca* and *Orobanche crenata* parasitizing parsley and *O. crenata* parasitizing faba bean, pea, lentil, vetch, celery, carrot, and glyphosate-resistant tomato, without causing damage to the host (Kasasian, 1973; Jacobsohn and Kelman, 1980; Mesa-García and García-Torres, 1985; Arjona-Berral et al., 1988; Goldwasser et al., 2003; Shilo et al., 2016). Therefore, the parasite is probably more sensitive than those hosts to glyphosate (Goldwasser and Kleifeld, 2004). To test this assumption, we studied the influence of glyphosate embedded in the growth medium on *P. aegyptiaca* callus. When glyphosate blocks EPSPS, S3P accumulates rapidly and is stored in cell protoplasts. It is then dephosphorylated to shikimic acid (3R,4S,5R-trihydroxy-1-cyclohexene-1-carboxylic acid) by vascular phosphorylases, instead of being further processed to chorismate and ultimately to aromatic amino acids and their derivatives (Binarova et al., 1994; Ulanov et al., 2009). Consequently, shikimate accumulation in plant tissues may serve as an indirect indication of inhibition by glyphosate (Holländer-Czytko and Amerhein, 1983; Binarova et al., 1994; Scheiber et al., 2006). Applied to *P. aegyptiaca* callus, glyphosate blocked EPSPS activity, and accumulation of the precursor shikimic acid was positively correlated with glyphosate concentration (**Figure 6**). Accordingly, shikimate accumulation in *P. aegyptiaca* parasitizing glyphosate-resistant tomato following glyphosate application has been recently shown (Shilo et al., 2016).

Glyphosate application resulted in an increase in total free amino acid content with a transient decrease in the proportion of aromatic amino acids (**Table 4** and Supplementary Figure 4). These results correspond well with Jaworski (1972) who reported a general increase in total free amino acids caused by glyphosate, and suggested that Phe depletion causes a reduction of protein biosynthesis. An increased free amino acid pool after ALS and EPSPS treatment was detected by Orcaray et al. (2010). It was also shown that although protein synthesis takes place after ALS inhibitor treatment, it does not contain newly incorporated nitrogen; all of the nitrogen is mainly scavenged from protein degradation (Zabalza et al., 2006). At the rate of 5 μM, glyphosate decreased the total content of free aromatic amino acids in liquid growth medium (**Table 5**). This might be due to cell death and release of their contents to the medium. Metabolic changes in the callus in the presence of glyphosate (reduction of aromatic amino acid biosynthesis and accumulation of shikimic acid) indicated that EPSPS was indeed inhibited in the parasite, as has been previously described in various plant species (Becerril et al., 1989; Bentley, 1990; Franz et al., 1997; Scheiber et al., 2006).

These results suggested that EPSPS is present and active in the parasite. However, only the existence of enzyme activity in parasite tissues can serve as direct proof of its having its own machinery for aromatic amino acid biosynthesis. Indeed, EPSPS activity was found in an *in vitro* assay in *P. aegyptiaca* flowering shoots and callus extracts for both the enzyme substrate S3P and its precursor shikimate. Although the natural substrate of EPSPS is S3P, the enzyme may also utilize shikimate as a substrate; however, this last reaction is less sensitive to glyphosate (Priestman et al., 2005) and indeed, in our experiments, ID_50_ for EPSPS extracted from the callus with S3P as substrate was 100 times lower than the same reaction using shikimate as the substrate (**Figure 7**).

Glyphosate inhibition of callus EPSPS activity and as a consequence, callus growth, indicates a direct influence of this herbicide on *P. aegyptiaca* enzyme activity and the requirement of this enzyme for growth and survival of the parasite. The data collected in this study indicate the presence of a pathway for aromatic amino acid biosynthesis in *P. aegyptiaca*, with EPSPS as an indicator for this pathway.

## Conclusion

Our main conclusion from this work is that *P. aegyptiaca* has the ability to synthesize branched-chain and aromatic amino acids through the activity of ALS and EPSPS, respectively. This is the first report to provide strong evidence for such activities and is based on the following observations: (i) putative *ALS* and *EPSPS* genes with ∼80% homology to their counterparts in *A. thaliana*; (ii) translocation of ALS-inhibiting herbicides and glyphosate applied to the host to *P. aegyptiaca*, causing the latter’s death; (iii) inhibited development of *P. aegyptiaca* shoots parasitizing tomato by direct injection of ALS-inhibiting herbicides into the parasite without any visible damage to the host plant; (iv) effective control of *P. aegyptiaca* by ALS-inhibiting herbicides and glyphosate in tissue culture; (v) ALS and EPSPS activity in *P. aegyptiaca* shoots and tissue culture; (vi) effective inhibition of ALS and EPSPS extracted from *P. aegyptiaca* shoots and tissue culture by ALS inhibitors (imazapic, imazapyr, and rimsulfuron) and glyphosate, respectively. Overall, our data indicate that the main mechanism by which ALS-inhibiting herbicides and glyphosate control broomrapes is direct inhibition of the enzymes ALS and EPSPS which are present and active in the broomrape tissues.

## Author Contributions

ED planned the study, analyzed and interpreted the data, performed the experiments, conducted the statistical analysis, drafted the manuscript, and ultimately approved the version to be published. SG contributed to the conception of the work, analyzed the data, and edited the manuscript. ES conducted experiments. YH performed experiments and contributed to the data analysis. RA contributed to the design of the work and to data interpretation, and edited the manuscript. JH planned the study, analyzed and interpreted the data, and drafted and edited the manuscript.

## Conflict of Interest Statement

The authors declare that the research was conducted in the absence of any commercial or financial relationships that could be construed as a potential conflict of interest.

## Footnotes

1 www.agilent.com

2 http://ppgp.huck.psu.edu

3 http://www.cbs.dtu.dk/services/ChloroP/

4 http://www.ebi.ac.uk/Tools/msa/muscle/

## Abbreviations

ALS: Acetolactate synthase
EPSPS: 5-Enolpyruvylshikimate-3-phosphate synthase
