# Supplementary material for: The Effects of Herbicides Targeting Aromatic and Branched Chain Amino Acid Biosynthesis Support the Presence of Functional Pathways in Broomrape
Source: Front Plant Sci. 2017 May 4;8:707. doi: 10.3389/fpls.2017.00707 (PMC5415608; doi:10.3389/fpls.2017.00707)

Supplementary Fig. 1. Effect of ALS inhibitors imazapic and imazapyr on the number of *P. aegyptiaca* flowering shoots developed on tomato plants resistant to ALS-inhibiting herbicides (HRT). Imazapic (38.4 g a.i. ha^{-1}) was applied on HRT plants at 277 growing degree days. Non-treated plants were used as a control. The number of broomrape shoots above soil level was counted at 3-day intervals. The experiment was conducted twice with 10 replicates. Comparison of the two experiments was performed using Fisher's *t*-test and the data were combined due to homogeneity of the variances. Vertical lines indicate LSD for specific observation dates at α = 0.05.

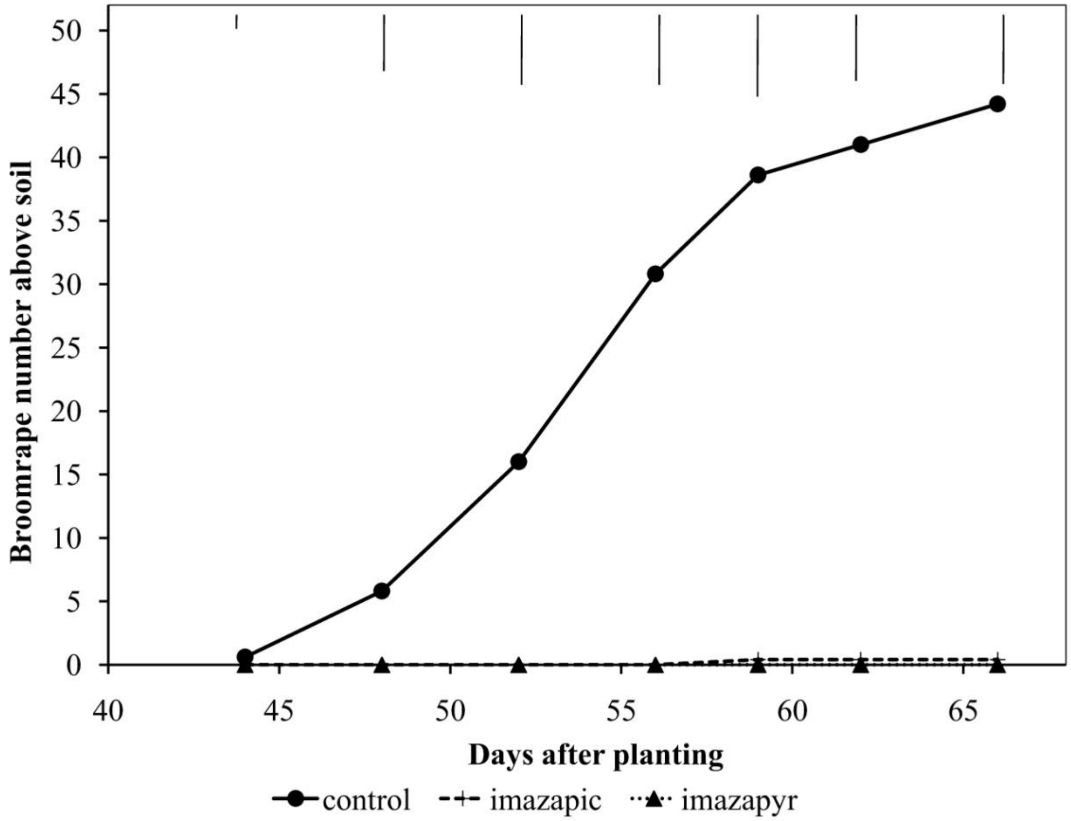

Supplement: Supplementary file 1 [file Image_1.PDF]
