# Supplementary material for: The Effects of Herbicides Targeting Aromatic and Branched Chain Amino Acid Biosynthesis Support the Presence of Functional Pathways in Broomrape
Source: Front Plant Sci. 2017 May 4;8:707. doi: 10.3389/fpls.2017.00707 (PMC5415608; doi:10.3389/fpls.2017.00707)

Supplementary Fig. 2 Effect of ALS-inhibiting herbicides injected into *P. aegyptiaca* shoots on their growth. When young broomrape shoots began to emerge above soil level (shoot height about 1 cm and 2–4 mm in diam.), they were injected with 5 µl of water (control), or 10 nmol of imazamox, imazapic, imazapyr, or sulfosulfuron. The injected broomrape shoots' height was evaluated on a daily basis. The results were subjected to ANOVA. Data were compared by LSD, on the basis of Tukey–Kramer Honestly Significant Difference test (α = 0.05). Vertical lines indicate LSD for specific dates. Results of the two experiments (10 replicates) were compared by Fisher's *t*-test and the data were combined due to homogeneity of the variances.

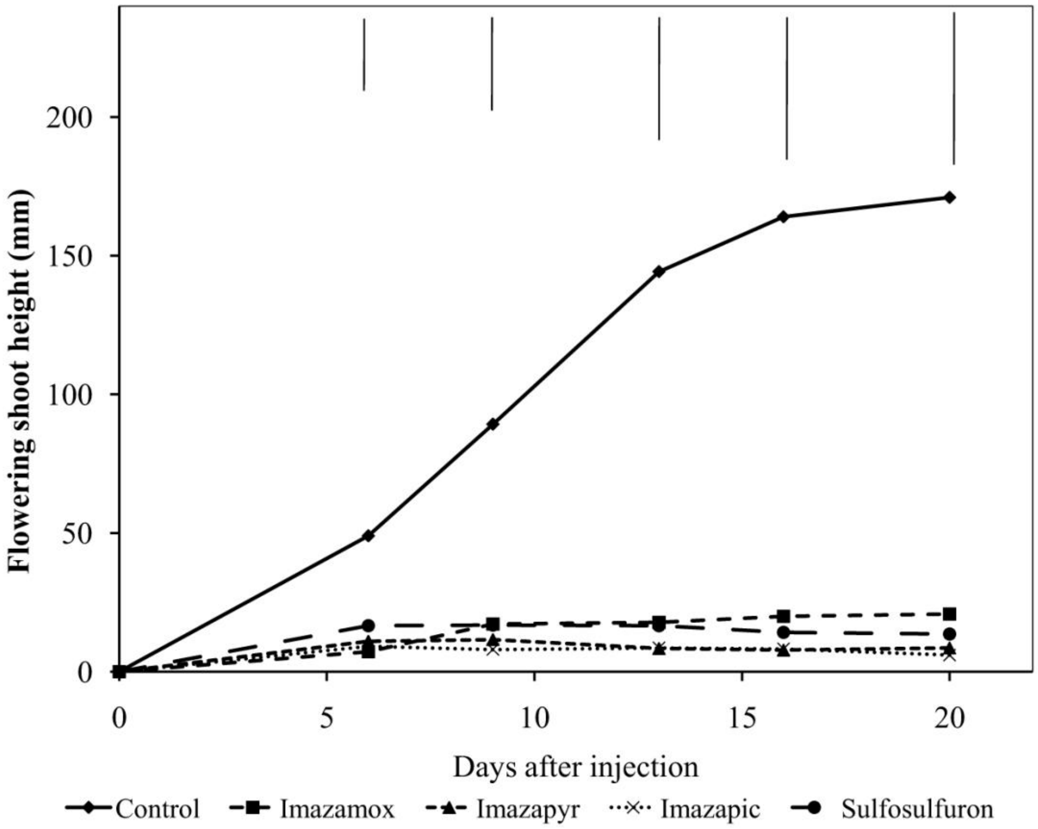

Supplement: Supplementary file 2 [file Image_2.PDF]
