# Supplementary material for: The Effects of Herbicides Targeting Aromatic and Branched Chain Amino Acid Biosynthesis Support the Presence of Functional Pathways in Broomrape
Source: Front Plant Sci. 2017 May 4;8:707. doi: 10.3389/fpls.2017.00707 (PMC5415608; doi:10.3389/fpls.2017.00707)

Supplementary Fig. 3 Influence of imazapic on the content of free valine (A), leucine (B) and isoleucine (C) in *P. aegyptiaca* calli grown in BCGM. The results were subjected to ANOVA by means of JMP Software, version 5.0 (SAS Institute Inc., Cary, NC, USA). Data were compared by least-significant differences (LSD), on the basis of Tukey–Kramer Honestly Significant Difference test (α = 0.05). Various letters indicate significant differences between treatments.

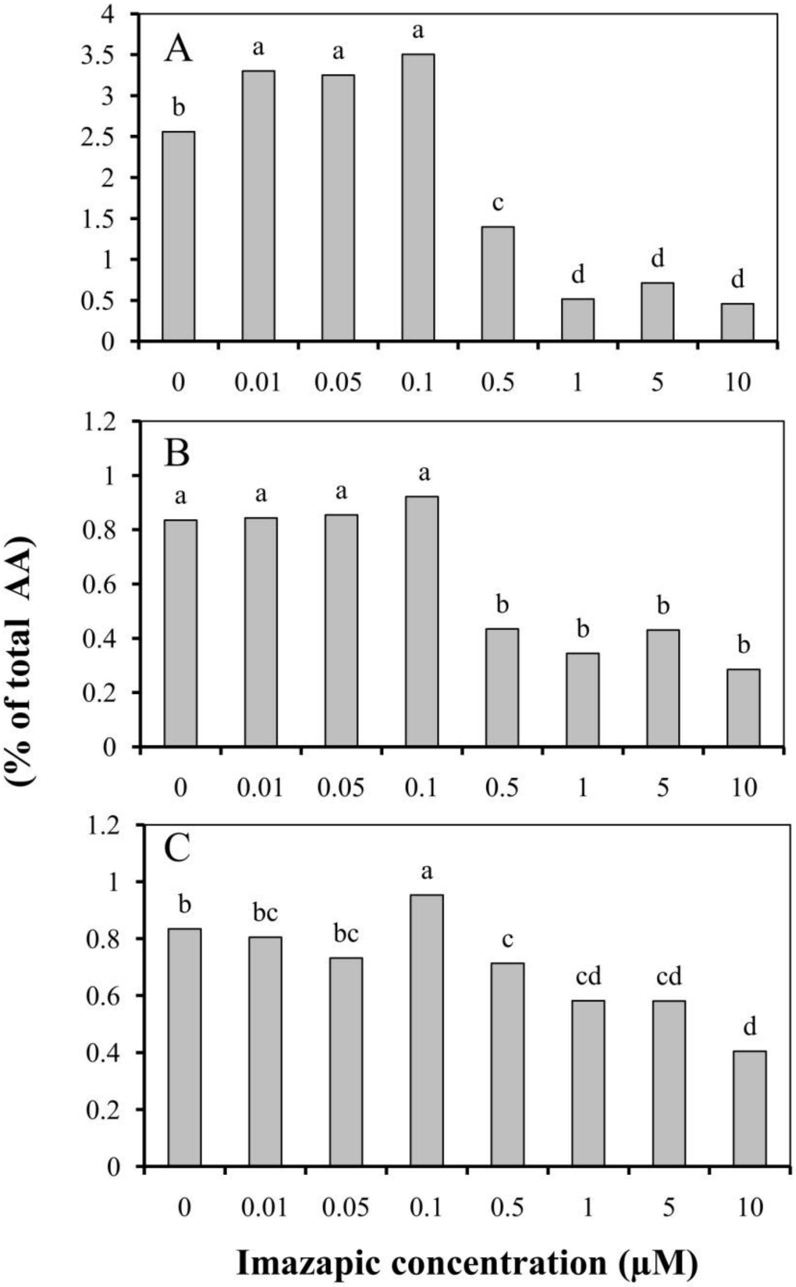

Supplement: Supplementary file 3 [file Image_3.PDF]
