# Supplementary material for: The Effects of Herbicides Targeting Aromatic and Branched Chain Amino Acid Biosynthesis Support the Presence of Functional Pathways in Broomrape
Source: Front Plant Sci. 2017 May 4;8:707. doi: 10.3389/fpls.2017.00707 (PMC5415608; doi:10.3389/fpls.2017.00707)

Supplementary Figure 4. Influence of glyphosate on the content of free Phe (A), Trp (B) and Tyr (C) in *P. aegyptiaca* callus grown in BCGM. Results were subjected to ANOVA using JMP Software, version 5.0. Data were compared by LSD, on the basis of Tukey–Kramer Honestly Significant Difference test ($\alpha = 0.05$). Different letters indicate significant differences between treatments.

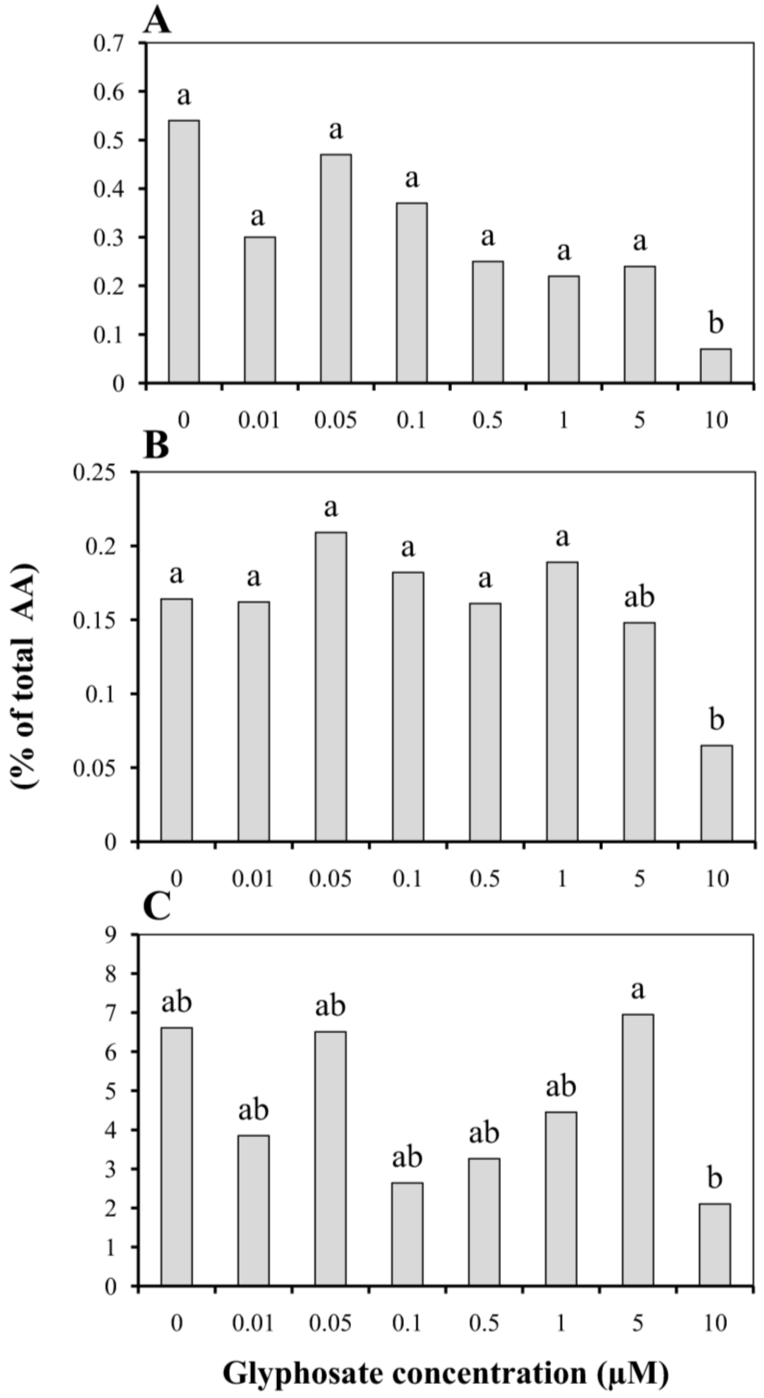

Supplement: Supplementary file 4 [file Image_4.PDF]
