# Supplementary material for: The Effects of Herbicides Targeting Aromatic and Branched Chain Amino Acid Biosynthesis Support the Presence of Functional Pathways in Broomrape
Source: Front Plant Sci. 2017 May 4;8:707. doi: 10.3389/fpls.2017.00707 (PMC5415608; doi:10.3389/fpls.2017.00707)

Supplementary Figure 5. Shikimic acid concentration in *P. aegyptiaca* callus and in liquid BCGM containing 5 µM glyphosate. The experiments was conducted with 3 replicates. The results were subjected to ANOVA by means of JMP Software, version 5.0. Data were compared by LSD, on the basis of Tukey–Kramer Honestly Significant Difference test (α = 0.05). Various letters indicate significant differences between various observations of the same treatment.

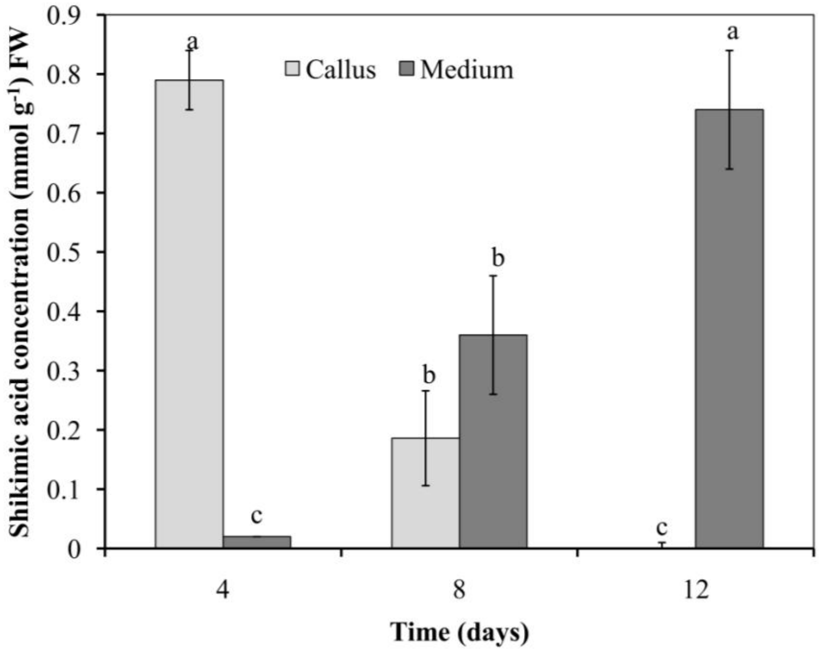

Supplement: Supplementary file 5 [file Image_5.PDF]
